# NMR and computational data of two novel antimicrobial peptides

**DOI:** 10.1016/j.dib.2016.06.009

**Published:** 2016-06-16

**Authors:** Lucia Falcigno, Gianna Palmieri, Marco Balestrieri, Yolande T.R. Proroga, Angelo Facchiano, Alessia Riccio, Federico Capuano, Raffaele Marrone, Giuseppe Campanile, Aniello Anastasio

**Affiliations:** a Department of Pharmacy, University of Naples Federico II, Via Mezzocannone, 16, 80134 Naples, Italy; b Institute of Biosciences and BioResources (IBBR)-UOS Na, National Research Council (CNR-IBBR),Via Pietro Castellino 111, 80131 Naples, Italy; c Department of Food Microbiology, Istituto Zooprofilattico Sperimentale del Mezzogiorno, Via della salute, 2, 80055 Portici, Italy; d Institute of Food Science National Research Council (CNR-ISA), Via Roma 52, 83100 Avellino, Italy; e Department of Veterinary Medicine and Animal Production, University of Naples Federico II, Via Federico Delpino 1, 80137 Naples, Italy

## Abstract

Here we report details on the design and conformational analysis of two novel peptides showing antimicrobial properties, as reported in the research article, “New antimicrobial peptides against foodborne pathogens: from in silico design to experimental evidence” G. Palmieri, M. Balestrieri, Y.T.R. Proroga, L. Falcigno, A. Facchiano, A. Riccio, F. Capuano, R. Marrone, G. Campanile, A. Anastasio (2016) [1]. NMR data, such as chemical shifts in two different solvents as well as aCH protons deviations from random coil values and NOE patterns, are shown together with the statistics of structural calculations. Strategy and particulars of molecular design are presented.

**Specifications Table**

**Table:** 

Subject area	*Chemistry*
More specific subject area	*Structural analysis*
Type of data	*Tables, graphs*
How data was acquired	*NMR (Varian Inova 600, equipped with a cryoprobe, and Varian Inova 400)*
Data format	*Analyzed*
Experimental factors	*Peptide solutions in DMSO-d*_*6*_*and in TFE-d*_*3*_*/H*_*2*_*O 50:50 (v/v)*
Experimental features	*Molecular modeling and peptide design.*
	*Acquisition and analysis of 1D and 2D NMR spectra of antimicrobial peptides to obtain NMR parameters, essentially NOE effects, used for molecular structures calculations by computational programs.*
Data source location	*Dept. of Pharmacy, University Federico II of Naples, Naples, Italy and Institute of Food Science National Research Council (CNR-ISA), Avellino, Italy*
Data accessibility	*Data is with this article*

**Value of the data**

• These data details the molecular design and NMR characterization of two novel antimicrobial peptides.
• NMR parameters, such as chemical shifts, in two different media can be useful for comparison with other peptides showing antimicrobial activities.
• The structural features emerging from in silico analysis and peptide molecular models can used to guide the design of analogs with enhanced biological activities.
• This data may provide insights for development of MTP-derived antimicrobials for food safety.

## Data

**Fig. 1:**
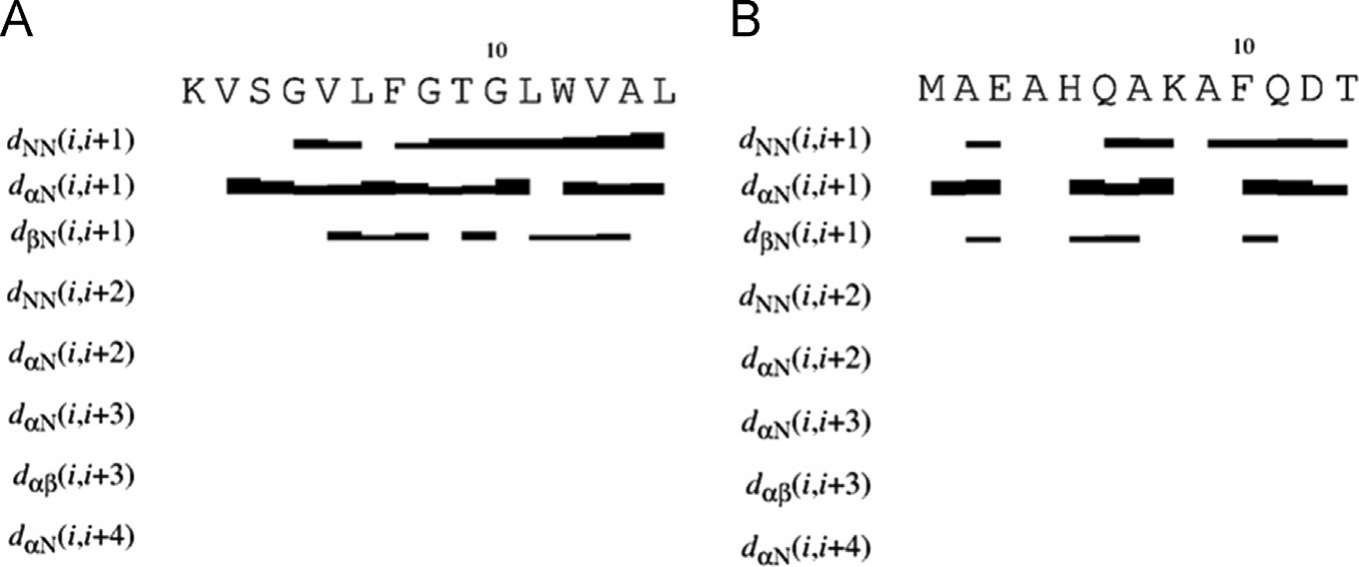
Relevant NOE contacts in DMSO for (**A**) MTP1 and (**B**) MTP2.

**Fig. 2:**
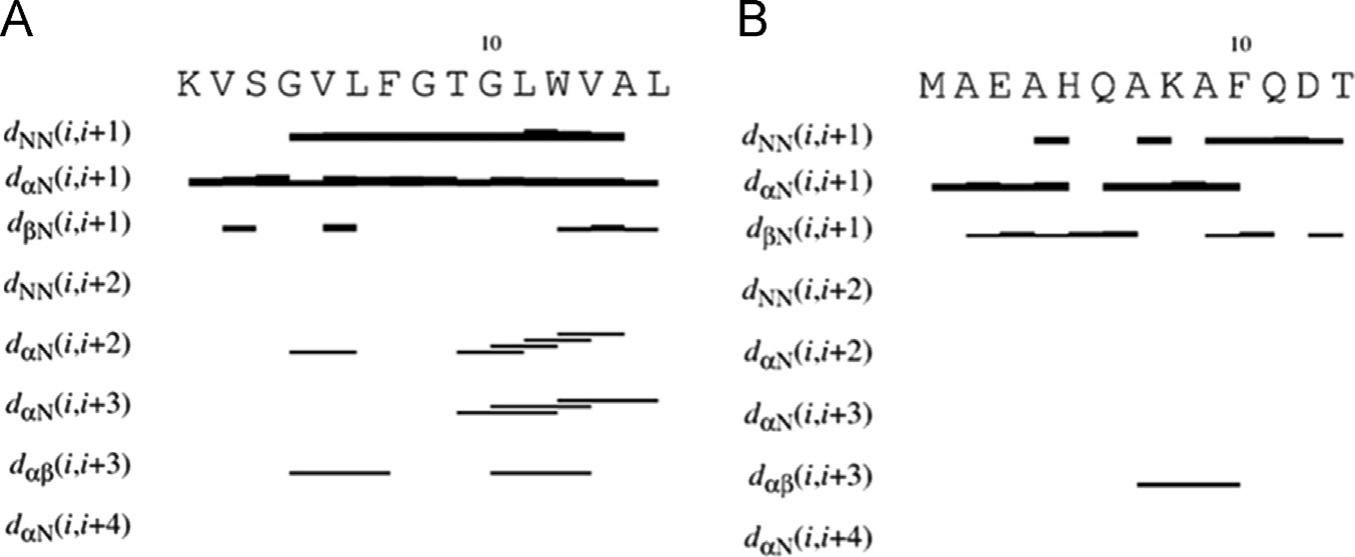
Relevant NOE contacts in TFE:H_2_O 1:1 for (**A**) MTP1 and (**B**) MTP2.

**Fig. 3:**
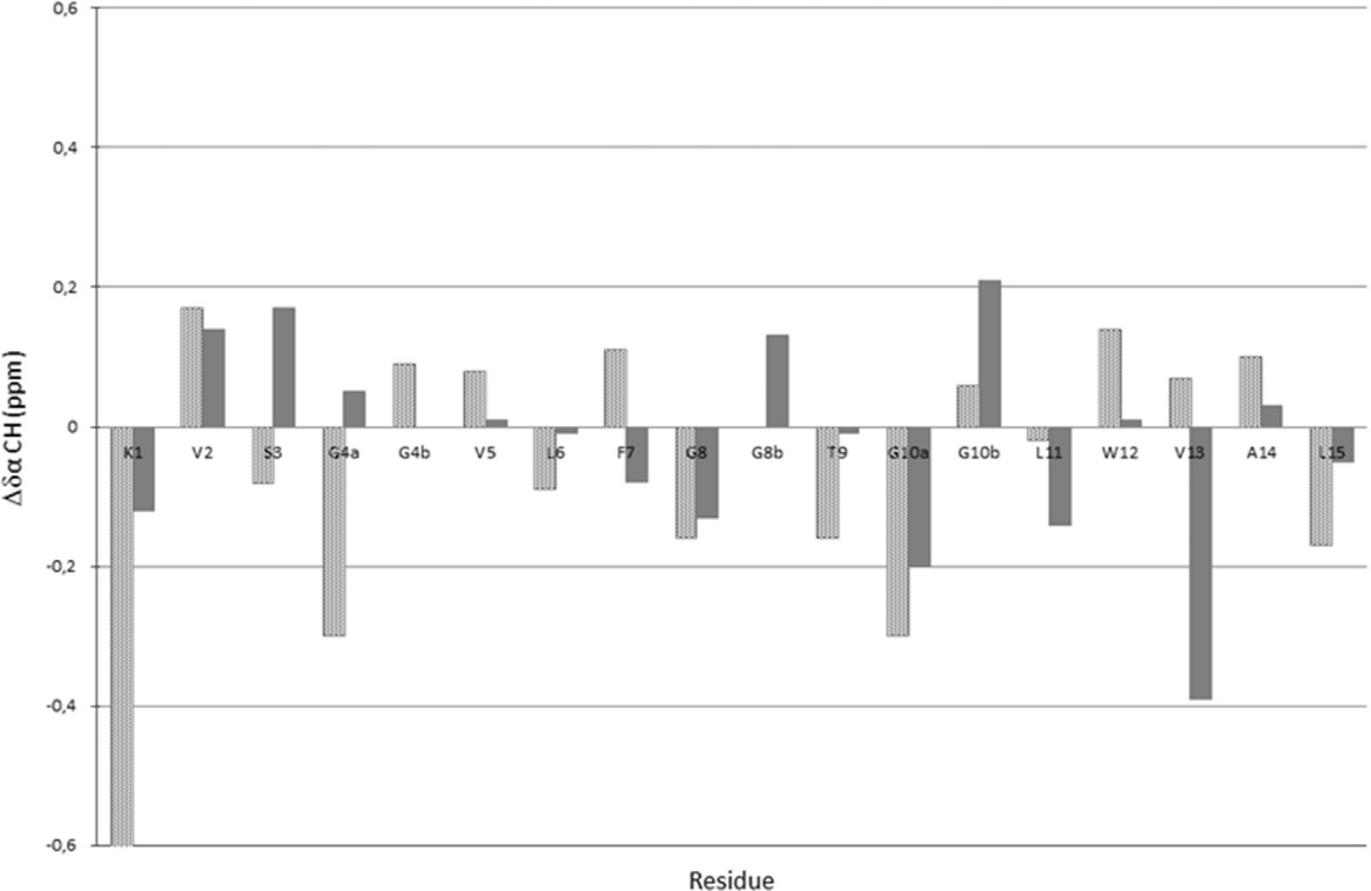
Comparison of deviations of αCH proton chemical shifts from random coil values [8] for MTP1 in DMSO (dotted bars) and TFE/H_2_O 1:1 (gray bars).

**Fig. 4:**
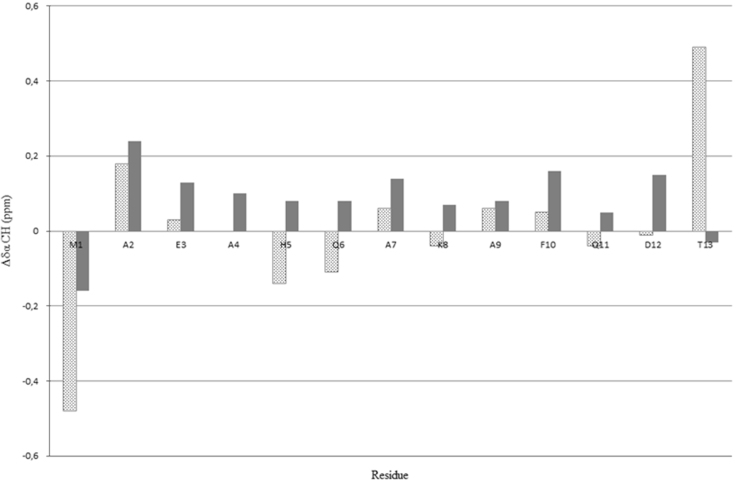
Comparison of deviations of αCH proton chemical shifts from random coil values [8] for MTP2 in DMSO (dotted bars) and TFE/H_2_O 1:1 (gray bars).

Data reported in the following are distinguished in three sub-sections: NMR analysis; computational methods; peptide design. In the first we report the proton chemical shifts of MTP1 and MTP2 peptides in DMSO and TFE/H_2_O 1:1 (Table 1, Table 2, Table 3, Table 4), together with the diagrams of the most relevant NOE effects (Fig. 1, Fig. 2) and the deviations of the αCH protons from random coil values (Fig. 3, Fig. 4). Next, we show the structural statistics of the molecular model calculations for MTP1 and MTP2 (Table 5, Table 6). Finally, the computed parameters from the computational tools used in the peptide designing.

## 1 Experimental design, materials and methods

### 1.1 NMR analysis

Two-dimensional (2D) experiments, such as total correlation spectroscopy (TOCSY) [2], nuclear Overhauser effect spectroscopy (NOESY) [3], and double quantum-filtered correlated spectroscopy (DQFCOSY) [4] were recorded by the phase sensitive States–Haberkorn method [5] on MTP1 and MTP2. TOCSY experiments were acquired with a 70 ms mixing time, while NOESY experiments were acquired with 150 and 300 ms mixing times; the water resonance was suppressed by using gradients [6].

Proton sequential assignments of the amino acid spin systems, obtained following the standard method proposed by Wuthrich [7], are reported in Table 1, Table 2, Table 3, Table 4.

In Fig. 1, Fig. 2 the structurally relevant NOE effects, observed for MTP1 and MTP2 in DMSO and TFE/H_2_O 1:1 are showed.

To compare the behavior of MTP1 and MTP2 peptides in the two different solvent systems, the αCH proton chemical shift deviations from random coil values [8] can be chosen as an useful reference (Fig. 3, Fig. 4).

Negative deviations of αCH proton chemical shift from random coil values <−0.1 ppm are indicative of helical structures, whilst deviations ranging from +1 to −1 point to random coil conformations [8].

### 1.2 Computational methods

Structure calculations for MTP1 and MTP2 performed by the standard CYANA simulated annealing schedule [9] were carried out by using NMR data evaluated in H_2_O/TFE-d_3_ 1:1, as reported in [1]. Statistical data of calculations are reported in Table 5, Table 6.

### 1.3 Peptide design

Prediction of antimicrobial activity has been performed by using the Computational tools at the Antimicrobial Peptide Database web site (http://aps.unmc.edu/AP/). Table 7, Table 8 report the different parameters computed. The potential antimicrobial activity prediction tool is evaluated by the protein-binding potential, or Boman index [10], obtained by meaning the free energy for transfer from cyclohexane to water, with ± inversion, on the basis of the amino acids composition of the peptide.

## Figures and Tables

**Table 1:** Proton chemical shifts (ppm) of MTP1 in DMSO-d_6_ at 298 K^a^.

Residue	**NH**	**αCH**	**βCH**	**γCH**	**Others**
**Lys**^**1**^	–	3.51	1.63	1.34	δCH_2_ 1.52; εCH_2_ 2.74; εNH 7.21
**Val**^**2**^	8.18	4.28	2.02	0.84	
**Ser**^**3**^	8.11	4.30	3.57	γOH 5.02	
**Gly**^**4**^	8.06	3.81; 3.74			
**Val**^**5**^	7.76	4.19	1.91	0.74	
**Leu**^**6**^	7.98	4.26	1.36	1.49	δCH_3_ 0.82
**Phe**^**7**^	7.92	4.53	3.02, 2.84		
**Gly**^**8**^	8.24	3.81			
**Thr**^**9**^	7.81	4.21	4.08	γCH_3_ 1.06 γOH 4.96	
**Gly**^**10**^	8.08	3.81; 3.71			
**Leu**^**11**^	7.87	4.33	1.39	1.51	δCH_3_ 0.82
**Trp**^**12**^	8.13	4.56	3.14; 2.96		2H 7.11; 4H 7.30; 5H 7.04; 6H 6.95; 7H 7.54; NH 10.74
**Val**^**13**^	7.72	4.18	1.94	0.83	
**Ala**^**14**^	8.02	4.29	1.19		
**Leu**^**15**^	7.72	4.18	1.43	1.58	δCH_3_ 0.82 CONH_2_ ter 7.24, 6.95

a Chemical shifts were referred to DMSO (2.5 ppm).

**Table 2:** Proton chemical shifts (ppm) of MTP1 in TFE-d_3_:H_2_O 1:1 at 298 K^a^.

Residue	**NH**	**αCH**	**βCH**	**γCH**	**Others**
**Lys**^**1**^	–	4.11	1.98	1.51	δCH_2_ 1.76; εCH_2_ 3.06; εNH -
**Val**^**2**^	8.45	4.25	2.12	1.01	
**Ser**^**3**^	8.16	4.55	3.95, 3.88		
**Gly**^**4**^	8.24	4.02			
**Val**^**5**^	7.76	4.12	2.08	0.95	
**Leu**^**6**^	7.84	4.34	1.62	1.51	δCH_3_ 0.88
**Phe**^**7**^	7.78	4.34	3.10, 3.01		HD 7.24, HE 7.06
**Gly**^**8**^	7.80	3.98, 3.78			
**Thr**^**9**^	7.79	4.36	4.36	γCH_3_ 1.30	
**Gly**^**10**^	8.11	3.91; 3.86			
**Leu**^**11**^	7.74	4.21	1.59	1.59	δCH_3_ 0.92
**Trp**^**12**^	7.60	4.43	3.35		2H 7.26; 4H 7.46; 5H 7.26; 6H 7.15; 7H 7.57; NH 9.82
**Val**^**13**^	7.40	3.72	2.00	0.85	
**Ala**^**14**^	7.69	4.22	1.45		
**Leu**^**15**^	7.80	4.30	1.78	1.62	δCH_3_ 0.89 CONH_2_ ter 7.13, 6.75

a Chemical shifts were referred to internal sodium 3-(trimethylsilyl) propionate 2,2,3,3-d4 (TSP).

**Table 3:** Proton chemical shifts (ppm) of MTP2 in DMSO-d_6_ at 298 K^a^.

Residue	NH	αCH	βCH	γCH	Others
**Met^1^**	–	3.84	1.96	2.51	S-CH_3_ 2.05
**Ala^2^**	8.62	4.37	1.24		
**Glu^3^**	8.15	4.27	1.89, 1.73	2.25	
**Ala^4^**	7.88	4.19	1.16		
**His^5^**	8.12	4.45	2.91		2H 4H
**Gln^6^**	8.03	4.17	1.87, 1.72	2.08	7.27, 6.79
**Ala^7^**	8.38	4.25	1.21		
**Lys^8^**	7.90	4.19	1.62	1.28	δCH_2_ 1.48; εCH_2_ 2.74; εNH 7.64
**Ala^9^**	8.08	4.25	1.19		
**Phe^10^**	7.94	4.47	3.04; 2.81		7.23
**Gln^11^**	8.09	4.24	1.84, 1.77	2.10	7.25, 6.79
**Asp^12^**	8.33	4.62	2.74, 2.56		
**Thr^13^**	7.51	4.86	4.06	γCH_3_ 1.00 γOH 4.86	CONH_2_ ter 7.15

a Chemical shifts were referred to DMSO (2.5 ppm).

**Table 4:** Proton chemical shifts (ppm) of MTP2 in H_2_O/TFE-d_3_ 1:1^a^.

Residue	**NH**	**αCH**	**βCH**	**γCH**	**Others**
**Met**^**1**^	–	4.16	2.24	2.68	S-CH_3_ 2.17
**Ala**^**2**^	8.61	4.43	1.45		
**Glu**^**3**^	8.36	4.37	2.13, 2.00	2.47	
**Ala**^**4**^	8.20	4.29	1.39		
**His**^**5**^	8.36	4.67	3.33, 3.24		2H 8.59 4H 7.31
**Gln**^**6**^	8.31	4.36	2.15, 2.05	2.39	δCH_2_ 7.41, 6.72
**Ala**^**7**^	8.24	4.33	1.45		
**Lys**^**8**^	8.04	4.30	1.84	1.48	δCH_2_ 1.77; εCH_2_ 3.03; εNH
**Ala**^**9**^	8.02	4.27	1.34		
**Phe**^**10**^	7.90	4.58	3.21; 3.12		7.28
**Gln**^**11**^	8.11	4.33	2.14, 2.05	2.36	δCH_2_ 7.39, 6.69
**Asp**^**12**^	8.30	4.78	2.97, 2.86		
**Thr**^**13**^	7.93	4.34	4.34	γCH_3_ 1.26	CONH_2_ ter 7.48, 7.02

a Chemical shifts were referred to internal sodium 3-(trimethylsilyl) propionate 2,2,3,3-d4 (TSP).

**Table 5:** CYANA Structural Statistics of MTP1 in TFE/H_2_O 1/1.

	NMR restraints
Distance restraints	111
Intraresidue	60
Sequential (|*i*−*j*| = 1)	37
Medium-range (1< |*i*−*j*| ≤ 4)	14
Torsion angle restraints	4
	Violation statistics (100 structures)
CYANA TF (Å^2^)	1.11 ± 1.07 Å^2^
	Residual distance constraint violations (Å)
Number > 0.2 Å	0
	Angle constraint violations (°)
Number > 5.0°	0
Mean global backbone RMSD	2.92 ± 0.59 Å
Mean global heavy RMSD	4.02 ± 0.51 Å
	Violation statistics (40 structures)
CYANA TF (Å^2^)	0.34 ± 6.43E-02 Å^2^
	Residual distance constraint violations (Å)
Number > 0.2 Å	0
	Angle constraint violations (°)
Number > 5.0°	0
Mean global backbone RMSD	2.71 ± 0.61 Å
Mean global heavy RMSD	3.86 ± 0.48 Å

**Table 6:** CYANA structural statistics of MTP2 in TFE/H_2_O 1/1.

	NMR restraints
Distance restraints	92
Intraresidue	62
Sequential (|*i*−*j*| = 1)	28
Medium-range (1< |*i*−*j*| ≤ 4)	2
Torsion angle restraints	3
	Violation statistics (100 structures)
CYANA TF (Å^2^)	4.99E-02 ± 6.69E-02 Å^2^
	Residual distance constraint violations (Å)
Number > 0.2 Å	0
	Angle constraint violations (°)
Number > 5.0°	0
Mean global backbone RMSD	2.74 ± 0.53 Å
Mean global heavy RMSD	4.03 ± 0.41 Å
	Violation statistics (40 structures)
CYANA TF (Å^2^)	6.37E-03 ± 3.26E-03 Å^2^
	Residual distance constraint violations (Å)
Number > 0.2 Å	0
Number > 5.0°	0
Mean global backbone RMSD	2.64 ± 0.50 Å
Mean global heavy RMSD	3.87 ± 0.54 Å

**Table 7:** Physicochemical properties of the 13-mer wild type (1–13 residues of the N-terminal tail of CPT-1a) and of hypothetical mutated peptides obtained by substitution of each amino acid with glycine. Amino acid position indicated in red resulted to be the most reactive in improving the potential antimicrobial activity.

Sequence	BI (kcal/mol)	APD (%)	Total net charge	GRAVY	W–W
MAEAHQAVAFQFT	0.42	61	−1	0.346	1.75
					
Substitution					
**G------------**	0.53	53	−1	0.169	1.99
**-G-----------**	0.49	53	−1	0.177	1.59
**--G----------**	−0.17	61	0	0.584	−0.26
**---G---------**	0.49	53	−1	0.177	1.59
**----G--------**	0	61	−1	0.561	1.59
**-----G-------**	−0.07	61	−1	0.585	1.18
**------G------**	0.49	53	−1	0.177	1.59
-------G-----	**0.66**	**53**	**−1**	**−0.007**	**1.69**
**--------G----**	0.49	53	−1	0.177	1.59
---------G---	**0.58**	**53**	**−1**	**0.1**	**2.89**
**----------G--**	−0.07	61	−1	0.546	1.18
-----------G-	**0.58**	**53**	**−1**	**0.1**	**2.89**
**------------G**	0.15	61	−1	0.369	1.62
**-------**K**-----**	**1.16**	**53**	**0**	−0.277	2.67
**-----------**D**-**	**1.32**	**53**	**−2**	−0.138	4.11
-------K---D-	**2.06**	46	−1	−0.761	5.03

BI, Boman index; APD, total hydrophobic ratiocharge; GRAVY, the Grand Average hydropathy value of the peptide; W–W, the Wimley-White whole-residue hydrophobicity of the peptide (i.e. the sum of whole-residue free energy of transfer of the peptide from water to POPC interface).

**Table 8:** Structural and physicochemical properties of MTP1 and MTP2.

Peptide	**Amino acid sequence**	**Mol weight**	**BI (kcal/mol)**	**APD (%)**	**Total net charge**	**GRAVY index**	**W–W**
MTP1	K**V**SG**VLF**GTG**LWVAL**	1546.90	−1.68	60	+1	1.41	2.99
MTP2	MAE**A**HQ**A**KA**F**QDT	1447.60	2.06	46	−1	−0.70	5.03

Underlined residues are hydrophobic; underlined residues in bold are both hydrophobic and located on the same peptide surface. BI, APD, GAVY, W–W see footnote in Table 7.
