# Supplementary material for: NMR and computational data of two novel antimicrobial peptides
Source: Data Brief. 2016 Jun 16;8:562–9. doi: 10.1016/j.dib.2016.06.009 (PMC4961720; doi:10.1016/j.dib.2016.06.009)
Supplement: Supplementary file 1 — Supplementary material [file mmc1.pdf]

Conflicts of Interest Statement

Manuscript title: *NMR and computational data of two novel antimicrobial peptides*

The authors whose names are listed immediately below certify that they have NO affiliations with or involvement in any organization or entity with any financial interest (such as honoraria; educational grants; participation in speakers' bureaus; membership, employment, consultancies, stock ownership, or other equity interest; and expert testimony or patent-licensing arrangements), or non-fi nancial interest (such as personal or professional relationships, affi liations, knowledge or beliefs) in the subject matter or materials discussed in this manuscript.

Author names: Lucia Falcigno, Gianna Palmieri, Marco Balestrieri, Yolande T. R. Proroga, Angelo Facchiano, Alessia Riccio, Federico Capuano, Raffaele Marrone, Giuseppe Campanile and Aniello Anastasio.

This statement is signed by all the authors to indicate agreement that the above information is true and correct:

Author's name	Author's signature	Date
Lucia Falcigno		1/06/2016
Gianna Palmieri		1/06/2016
Marco Balestrieri		01/06/2016
Yolande T. R. Proroga		1/06/2016
Angelo Facchiano		01/06/2016
Alessia Riccio		1/6/2016
Federico Capuano		1/06/2016
Raffaele Marrone		1/06/2016
Giuseppe Campanile		1/06/16
Aniello Anastasio		1/06/16
